# SDI-118, a novel procognitive SV2A modulator: First-in-human randomized controlled trial including PET/fMRI assessment of target engagement

**DOI:** 10.3389/fphar.2022.1066447

**Published:** 2023-01-17

**Authors:** Wouter Botermans, Michel Koole, Koen Van Laere, Jonathan R. Savidge, John A. Kemp, Stefan Sunaert, Maeve M. Duffy, Steven Ramael, Andrea M. Cesura, Kevin D’Ostilio, Denis Gossen, Torsten M. Madsen, Thomas Lodeweyckx, Jan de Hoon

**Affiliations:** ^1^ Center for Clinical Pharmacology, University Hospital Leuven, Leuven, Belgium; ^2^ Nuclear Medicine and Molecular Imaging, Imaging and Pathology, KU Leuven and University Hospital Leuven, Leuven, Belgium; ^3^ Syndesi Therapeutics SA, Louvain-LaNeuve, Belgium; ^4^ Translational MRI, Department of Imaging and Pathology, KU Leuven, Leuven Brain Institute, KU Leuven, Radiology, University Hospital Leuven, Leuven, Belgium; ^5^ Aepodia SA, Louvain-LaNeuve, Belgium

**Keywords:** randomized controlled trial, SDI-118, synaptic vesicle glycoprotein, SV2A, positron emission tomography, resting state functional MRI (rsfMRI), cognitive impairment (CI), target occupancy

## Abstract

**Background:** Current treatments for progressive neurodegenerative disorders characterized by cognitive impairment either have limited efficacy or are lacking altogether. SDI-118 is a small molecule which modulates the activity of synaptic vesicle glycoprotein 2A (SV2A) in the brain and shows cognitive enhancing effects in a range of animal models of cognitive deficit.

**Methods:** This first-in-human study evaluated safety, tolerability, and pharmacokinetics/pharmacodynamics of SDI-118 in single ascending oral doses up to 80 mg administered to 32 healthy male subjects. Brain target occupancy was measured in eight subjects using positron emission tomography with PET-ligand [^11^C]-UCB-J. Food effect was assessed in seven subjects. Mood state was regularly evaluated using standardized questionnaires, and resting state fMRI data were analyzed as exploratory objectives.

**Key Results:** At all doses tested, SDI-118 was well tolerated and appeared safe. Adverse events were mainly dizziness, hypersomnia, and somnolence. All were mild in intensity and increased in frequency with increasing administered dose. No dose-limiting adverse reactions were observed at any dose. SDI-118 displayed a linear pharmacokinetic profile with no significant food effect. Brain penetration and target engagement were demonstrated by a dose-proportional SV2A occupancy.

**Conclusion:** Single oral doses of SDI-118 up to 80 mg were very well tolerated in healthy male subjects. Dose-proportional SV2A occupancy in the brain was demonstrated with brain imaging. Adverse effects in humans mainly occurred in higher dose ranges, with high occupancy levels, and were all mild and self-limiting. These data support further clinical exploration of the compound in patients with cognitive disorders.

**Clinical Trial Registration:** https://clinicaltrials.gov/, identifier NCT05486195

## Introduction

Cognitive impairment is a hallmark of many central nervous system (CNS) disorders. This includes neurodegenerative disorders such as Alzheimer’s disease (AD) and other forms of dementia as well as psychiatric disorders such as schizophrenia and major depressive disorder. There is increasing evidence that synaptic dysfunction and loss underlie the cognitive impairment observed in these conditions. (Chen et al., 2018; Heurling et al., 2019; Holmes et al., 2019; Mecca et al., 2020; Onwordi et al., 2020; Michiels et al., 2021). The currently approved treatments for AD are limited to only two mechanisms of action: central inhibition of acetylcholinesterase (AChE) and *N*-methyl-d-aspartate (NMDA) receptor antagonism. Both mechanisms have limited efficacy and there is a significant medical need for alternative treatment approaches with improved efficacy to relieve symptoms of cognitive impairment. In addition, there are currently no approved treatments for cognitive impairment in many other disorders such as schizophrenia.

A new approach towards better cognitive functioning in these disorders is by improving synaptic transmission *via* modulation of the action of synaptic vesicle glycoprotein 2A (SV2A) in the presynaptic axon terminal. SV2A is an integral protein of presynaptic vesicles that is ubiquitously expressed in virtually all neuronal cell types. SV2A, through its interaction with synaptotagmin, plays a role in regulating calcium evoked vesicle fusion and thus neurotransmitter release *via* exocytosis. (Xu and Bajjalieh, 2001; Schivell et al., 2005; Custer et al., 2006; Chang and Südhof, 2009; Yao et al., 2010).

SV2A has been identified as the binding site for the antiepileptic drug levetiracetam. (Lynch et al., 2004). The more recently approved antiepileptic drug brivaracetam was developed as a compound displaying higher affinity binding to SV2A. (Malawska and KuligBrivaracetam, 2005). These molecules exert their effect by inhibiting high-frequency synaptic transmission in a use-dependent manner. (Yang and Rothman, 2009; Yang et al., 2015).

During the research program, a new class of compounds has been discovered that bind to SV2A without anticonvulsant activity but showing procognitive activity in animal models. (Löscher et al., 2016). SDI-118 is an orally available small molecule that shows high affinity for SV2A with a half maximal inhibitory concentration (IC_50_) of 13 nM in an *in vitro* radioligand binding study using human recombinant SV2A (hSV2A), and selectivity over other SV2 isoforms, SV2B (over 1000-fold) and SV2C (over 100-fold). (Lynch et al., 2004; Matagne et al., 2008). Unlike levetiracetam and brivaracetam, SDI-118 is without anti-convulsant effects despite good brain penetration and target occupancy, but showed cognitive enhancing effects in a range of animal models of cognitive deficit in rodents. Thus, it holds the potential for the treatment of cognitive disorders including AD and other forms of dementia, schizophrenia, and major depressive disorder.

Following preclinical regulatory toxicology and safety pharmacology studies performed in rats and dogs, the compound was brought into clinical development. The current manuscript provides a report of the first-in-human (FIH) single ascending (oral) dose (SAD) study with SDI-118 evaluating the safety and tolerability (primary endpoints), pharmacokinetic (PK) profile including characterization of a food effect (FE), and pharmacodynamics through target occupancy measurements using positron emission tomography (PET) in healthy male subjects (secondary endpoints). Resting state functional magnetic resonance imaging (rs-fMRI) data and alteration of mood state as assessed with questionnaires were also analyzed as exploratory endpoints.

## Materials and methods

This FIH study was conducted in accordance with International Council for Harmonization on Good Clinical Practice (ICH-GCP) requirements and the ethical principles that have their origin in the Declaration of Helsinki, and in compliance with the protocol approved by the Ethics Committee of the University Hospital of Leuven. Written informed consent was obtained from all participating subjects. All data were collected at the Center for Clinical Pharmacology and the Department of Nuclear Medicine of the University Hospital of Leuven, Belgium.

### Eligibility

Healthy male subjects between 18 and 50 years of age (inclusive) were eligible for inclusion, provided that they did not meet any of the following criteria: body mass index (BMI) outside of the range of 18–30 kg/m^2^, history or presence of any clinically significant illness or clinically significant abnormalities in vital signs, laboratory tests, electrocardiogram (ECG), physical and neurological examination, or use of concomitant medication or substances of abuse. Additionally, for the PET-MR part of the study, subjects were eligible if they did not have any contraindication for MRI-scanning or structural brain abnormalities as shown on the screening MRI-scan, contraindications for arterial line placement, or significant radiation exposure (i.e. exposure to radiation by a medical PET- or CT-device) in the last 12 months.

### Study design

The study consisted of three parts. Part A was a double-blind, placebo-controlled (in a 6:2 SDI-118:placebo ratio), single ascending oral dose study performed in 16 subjects equally divided into two cohorts. The number of subjects is classical for this type of study and no formal statistical hypothesis or testing had been considered. The randomisation list was prepared by the sponsor assigned statistician. No stratification was performed. Subjects were allocated to one of the two cohorts depending on their availability. Allocation numbers were assigned based on the order of screening. Dose levels of .3, 1, 3, 10, 30, 60, 90 and 120 mg of SDI-118 were set to be administered in an alternating panel design with a minimum washout period of 7 days between subsequent periods. In each period two different sentinel subjects were dosed (in a 1:1 SDI-118:placebo ratio) and followed-up for safety for at least 48 h before the rest of the cohort received the same dose level of the study drug. The starting dose of 0.3 mg was calculated to be below the maximum recommended starting dose (MRSD) as per EMA/FDA guidelines (FDA Guidance for Industry, 2005; EUROPEAN MEDICINES AGENCY, 2007) by a factor of approximately 500, based on a no observed adverse effect level (NOAEL) of 150 mg/kg/day in rats, determined as the most sensitive species. According to allometric scaling and SV2A occupancy simulations, and presuming PK linearity, a starting dose of 0.3 mg would provide a receptor occupancy in humans of approximately 5%. This was also below the minimum anticipated biological effect level (MABEL) in experimental rodent models which appeared to start at a SV2A occupancy of approximately 10%.

Part B had an open-label design in which up to six groups of two subjects received a single oral dose of SDI-118 as chosen from a range deemed safe and tolerable in Part A, and with PK data suggesting that a meaningful plasma exposure would be reached to occupy at least 10% of the SV2A protein target at T_max_ of SDI-118. During Part B PET-MR scans were taken to measure the target occupancy at two different time points after intake of SDI-118. This way the inclusion of up to 12 subjects provided a total of up to 24 potential data points. No statistical criteria were considered for the sample size of Part B.

Part C had an open-label 2-way crossover design in which 1 cohort of eight subjects was to be dosed on two occasions with the same dose level (as chosen from Part A and B), once in a fasting state and once after a standardized high-calorie, high-fat breakfast (with four subjects randomized to each arm per period) to assess the effect of food intake on drug absorption. With eight subjects randomized, the study should have at least 85% power to rule out any major food interaction. Powering was made assuming no mean difference between fed and fasted condition, and a within-subject coefficient of variation not larger than 45%.

SDI-118 was administered as a liquid formulation for the first dose level of 0.3 mg in Part A, and as a powder in capsule from 1 mg onwards.

### Safety

Safety and tolerability were assessed by questioning for adverse events (AEs), vital signs measurements (supine and orthostatic), 12-lead ECGs (always acquired in triplicate except during screening and post-study visit), standard lab safety tests on blood and urine samples, and physical and neurological examinations at regular time-points throughout the study.

### Pharmacokinetics

Blood samples for PK analysis were taken at regular time-points up to approximately 96 h post-dose in Part A and C. During the study, a protocol amendment was approved to extend the PK sampling scheme to include samples up to approximately 144 h post-dose, starting from the fourth dosing period. The rationale for this amendment was a longer than anticipated terminal elimination half-life of SDI-118 as concluded from preliminary PK data of the first three dosing periods. Plasma concentrations of SDI-118 were assayed using a GLP validated UPLC/MS-MS method. This method is linear for a range of concentrations from 0.200 to 500 ng/ml, with a lower limit of quantification (LLOQ) of 0.200 ng/ml. The following PK parameters were estimated: maximum plasma concentration C_max_, time of maximum plasma concentration T_max_, area under the plasma concentration *versus* time curve AUC, AUC_0-last_ (up to the last quantifiable concentration C_last_), AUC_inf_ (extrapolated to infinity), and apparent terminal elimination half-life t_½_. Dose proportionality of SDI-118 C_max_, AUC_0-last_, and AUC_inf_ from Part A was calculated based on a mixed-effect power model. Food effect on SDI-118 C_max_, AUC_0-last_, and AUC_inf_ from Part C was calculated using a 90% confidence interval (CI) on geometric mean ratios (fed/fasted) based on a mixed-effect analysis of variance (ANOVA) with median difference in T_max_ with 90% CI. Major interaction was excluded if the 90% CI on AUC and C_max_ ratios (fed/fasted) were within the 50%–200% boundaries. The food effect assessment in Part C was performed on a secondary population, i.e. the PK completers set, which excluded one subject who discontinued the study after one dosing period (cfr. infra).

### Pharmacodynamics

In Part B target occupancy in the brain was measured by PET-scans (GE Signa) with the PET-ligand [^11^C]-UCB-J (Nabulsi et al., 2016; Koole et al., 2019) at three different time points: pre-dose, around T_max_ (estimated on data collected in Part A), and approximately 24 h post-dose. [^11^C]-UCB-J was administered as an intravenous bolus into the antecubital vein over at least 15 s, followed by saline flush. An arterial line in the radial artery was placed in each subject to collect arterial blood samples to reconstruct a metabolite-corrected arterial plasma input function.

Dynamic PET scans were acquired in 3D list-mode for 90 min. List-mode data were rebinned into 27 time frames with a gradually increasing frame duration (6 × 15 s—3 × 30 s—3 × 60 s—2 × 90 s—2 × 3 min—9 × 5 min—3 × 10 min) and corrected for deadtime, randoms, and scatter. To correct for attenuation, a Zero-Echo Time (ZTE) scan was acquired simultaneously with the PET data to generate a subject-specific, MR-based attenuation map. Each frame was reconstructed with an iterative algorithm (OSEM—Ordered Subsets Expectation Maximization, four iterations, 28 subsets), modeling detector response, and using time of flight information (TOF). Each reconstructed frame consisted of 89 slices with a matrix size of 192 × 192, corresponding to an in-plane pixel size of 1.56 mm and a slice separation of 2.78 mm. Finally, smoothing was performed by applying a Gaussian filter (4.5 mm Full Width Half Maximum–FWHM) to each time frame.

SDI-118 SV2A occupancy was estimated globally using a full kinetic one-tissue compartmental modeling approach with an arterial input function (1TCM), and target occupancy was assessed with a Lassen plot using 13 main regions of interest: the overall cortex, the frontal, temporal, parietal and occipital cortex, the insula, the anterior and posterior cingulate cortex, the cerebellum, the striatum, the thalamus, the hippocampus, and the brainstem.

The PK/PD analysis set included all subjects from Part B with at least one available valid combined measurement of PK and PD, who received treatment and experienced no protocol deviations with relevant impact on PK or PD data. The global target occupancy based on the 1TCM method was analyzed as a function of the first PK concentration during the T_max_ and 24-h post-dose scans. To estimate the relationship between the observed concentration at the beginning of the PET measurement and the receptor occupancy, a mixed-effect maximal effective concentration (E_max_) model with two parameters E_max_ and half maximal effective concentration (EC_50_), with a random intercept was used. Parameter estimates, standard errors, and 95% confidence intervals were reported. Predictions from the model were reported with 95% confidence and prediction intervals to inform the required doses to produce these target occupancy values at T_max_ and 24-h post-dose.

In addition to the PET-data, resting state blood-oxygen-level-dependent (BOLD) fMRI measurements were simultaneously obtained in all subjects from Part B as an exploratory objective to measure the effect on resting state cerebral connectivity. BOLD fMRI was performed in interleave mode with a repetition time of 1.212 m, while using Hyperband 2). In total, 305 volumes were acquired per scan, with each volume consisting of 42 slices with a matrix size of 64 × 64. This corresponded to a voxel size of 3.44 × 3.44 × 4.00 mm^3^.

All fMRI data were processed with the CONN toolbox (release 19c) (Whitfield-Gabrieli and Conn, 2012) using a data driven approach. After standard pre-processing steps, a group-ICA (Independent Component Analysis) was applied to extract a predefined number of 32 independent components which were then spatially matched with a predefined set of functional networks comprising the Default Mode, SensoriMotor, Visual, Salience, Dorsal Attention, FrontoParietal, Language, and Cerebellar networks. Independent components which were clearly identified with a network were considered for further analysis and used for a seed-based connectivity analysis. For this second level connectivity analysis, a weighted General Linear Model (GLM) approach was used to make inferences about differences in functional connectivity between conditions on a seed basis using the nodes of the previously identified independent components as seed regions. To evaluate a dose-dependent effect, resulting beta values which represent the strength of functional connectivity between seed and target regions, were compared between baseline and all post dose conditions at T_max_.

As an exploratory objective in Part A and Part C, alteration of mood state was assessed through Bond and Lader Visual Analog Scale (BL-VAS) (Bond and Lader, 1974) and Profile of Mood States short form (POMS-SF) (Curran et al., 1995) questionnaires at regular time points throughout the study. Changes from baseline were calculated at each time point using analysis of covariance (ANCOVA).

## Results

### Subjects

Between 14 March 2019 and 28 June 2019, a total of 32 subjects meeting all eligibility criteria were enrolled of which 31 completed the study as per protocol. Demographic data of the enrolled subjects is displayed in Table 1. Follow-up of the last subject was completed on 29 July 2019. One subject participating in Part C withdrew his consent after the first study period for personal reasons. This subject did not receive the study drug in a fed state but was not replaced. The acquired data from this subject was included in both the safety and PK analysis sets but left out of the PK completers set.

**TABLE 1:** Demographic characteristics.

		Part A / SAD Cohort 1 (n=8)	Part A / SAD Cohort 2 (n=8)	Part B / PET Cohort 3 (n=8)	Part C / FE Cohort 4 (n=8)	All (n=32)
Age (years)	Mean	32.6	35.4	24.9	28.0	30.2
	95% CI	[26.1 ; 39.1]	[28.6 ; 42.2]	[20.9 ; 28.8]	[22.2 ; 33.8]	[27.4 ; 33.0]
	Median	33.5	34.5	23.5	24.5	28.0
	Min ; Max	22 ; 46	26 ; 48	19 ; 34	22 ; 40	19 ; 48
Race (%)	Black	1 (12.5%)	1 (12.5%)	0	0	2 (6.3%)
	White	7 (87.5%)	7 (87.5%)	8 (100%)	8 (100%)	30 (93.8%)

Demographic characteristics (age and race) of the included subjects. SAD, single ascending dose; PET, positron emission tomography; FE, food effect; CI, confidence interval.

The dose levels ultimately administered in Part A were 0.3, 1, 3, 10, 20, 40, 60, and 80 mg. Data generated during the study indicated a higher than anticipated target occupancy at lower doses, which led to the decision to include the 20 mg dose and to apply lower further dose increments.

In Part B four single doses (1, 2, 10, and 20 mg) of SDI-118 were tested in a total of eight subjects, with each dose level administered to two subjects. Dynamic [^11^C]-UCB-J PET scans were taken around T_max_ of SDI-118 (i.e. 2 h post-dose) and approximately 24 h post-dose. The mean activity of [^11^C]-UCB-J per injection was 257 ± 43 MBq (mean ± SD), with an injected molar activity of 67 ± 18 GBq/μmol (mean injected mass 1.3 µg), 64 ± 22 GBq/μmol (1.5 µg), and 60 ± 15 GBq/μmol (1.4 µg) at baseline, T_max_, and 24 h post-dose, respectively.

The dose level chosen for Part C was 10 mg in order to reach an adequate peak plasma concentration to occupy at least 50% of SV2A receptors (as observed in Part B).

### Safety and tolerability

The most frequently reported drug related TEAEs in all subjects (n = 32) after the administration of single doses of SDI-118 were dizziness (12 events by nine subjects), hypersomnia (6 events by five subjects; also reported once after placebo administration), and somnolence (2 events by two subjects). Somnolence only occurred at the highest administered dose of SDI-118 (80 mg). The onset time of these events was generally short after dose administration (between minutes and up to 4 h), and their duration was usually short (resolution within the same day or the day after at the most, in the case of hypersomnia). Other possibly drug related TEAEs were postural dizziness, headache, irritability (all were observed after placebo administration as well), blurred vision (1 account with short duration and no clear relationship with the investigational medicinal product), and fatigue (1 account at the 10 mg dose level). All drug related TEAEs were mild in intensity. Table 2 shows all possibly related TEAEs that occurred during the study pooled for all subjects. There were no serious adverse events (SAEs) and no subjects discontinued the study due to adverse events. There were no out-of-range values for vital signs, laboratory parameters, and ECG intervals that were considered related to the administration of SDI-118. No abnormalities were found upon neurological examination.

**TABLE 2:** Drug related treatment emergent adverse events (TEAEs).

	0.3 mg		1 mg		2 mg		3 mg		10 mg		20 mg		40 mg		60 mg			80 mg		Placebo			
	(N=6)		(N=8)		(N=2)		(N=6)		(N=16)		(N=8)		(N=6)		(N=6)			(N=6)		(N=16)	
System Organ Class Preferred Term	No.	N (%)	No.	N (%)	No.	N (%)	No.	N (%)	No.	N (%)	No.	N (%)	No.	N (%)	No.	N (%)		No.	N (%)	No.		N (%)	
All	-	-	1	1 (12.5)	-	-	1	1 (16.7)	5	4 (66.7)	4	4 (50.0)	4	3 (50.0)	4	3 (50.0)		8	4 (66.7)	4		3 (18.75)	
**Nervous System Disorders**	-	-	**1**	**1 (12.5)**	-	-	-	-	**3**	**3 (50.0)**	**4**	**4 (50.0)**	**4**	**3 (50.0)**	**3**	**2 (33.3)**		**7**	**4 (66.7)**	**2**		**2 (12.5)**	
Dizziness	-	-	-	-	-	-	-	-	1	1 (16.7)	4	4 (50.0)	3	3 (50.0)	2	2 (33.3)		2	2 (33.3)	-		-	
Hypersomnia	-	-	-	-	-	-	-	-	2	2 (33.3)	-	-	1	1 (16.7)	1	1 (16.7)		2	2 (33.3)	1		1 (6.25)	
Headache	-	-	1	1 (12.5)	-	-	-	-	-	-	-	-	-	-	-	-		1	1 (16.7)	1		1 (6.25)	
Somnolence	-	-	-	-	-	-	-	-	-	-	-	-	-	-	-	-		2	2 (33.3)	-		-	
**Psychiatric Disorders**	-	-	-	-	-	-	-	-	-	-	-	-	-	-	-	-		**1**	**1 (16.7)**	**1**		**1 (6.25)**	
Irritability	-	-	-	-	-	-	-	-	-	-	-	-	-	-	-	-		1	1 (16.7)	1		1 (6.25)	
**Eye Disorders**	-	-	-	-	-	-	-	-	**1**	**1 (16.7)**	-	-	-	-	-	-		-	-	-		-	
Blurred vision	-	-	-	-	-	-	-	-	1	1 (16.7)	-	-	-	-	-	-		-	-	-		-	
**Cardiac Disorders**	-	-	-	-	-	-	**1**	**1 (16.7)**	-	-	-	-	-	-	**1**	**1 (16.7)**		-	-	**1**		**1 (6.25)**	
Postural Dizziness	-	-	-	-	-	-	1	1 (16.7)	-	-	-	-	-	-	1	1 (16.7)		-	-	1		1 (6.25)	
**General Disorders**	-	-	-	-	-	-	-	-	**1**	**1 (16.7)**	-	-	-	-	-	-		-	-	-		-	
Fatigue	-	-	-	-	-	-	-	-	1	1 (16.7)	-	-	-	-	-	-		-	-	-		-	

TEAEs, as reported per dose level controlled against the pooled placebo group. TEAEs, were categorized as drug related if they were considered to be either possibly, probably, or definitely related to study drug intake by the investigator. No. = number of observations; N = number of subjects (per group).

In agreement with the low potential to inhibit hERG seen in preclinical *in vitro* studies, and the absence of any adverse findings in the cardiovascular safety pharmacology study in dogs, no QT-interval prolongations were observed, although no formal correlation was made between the results of ECG intervals and the plasma concentrations of SDI-118 obtained at the same time. No subject had a single QT-interval value above 500 m, or a single corrected QT-interval Fridericia (QTcF) value above 450 m. The largest observed mean increase from baseline QTcF was +6.1 ms (12 h post-dose at 60 mg).

Since actual washout periods were at least 12 days, the longer measured half-life than initially anticipated (cfr. infra) did not pose any risks for carry-over effects.

### Pharmacokinetics

**FIGURE 1:**
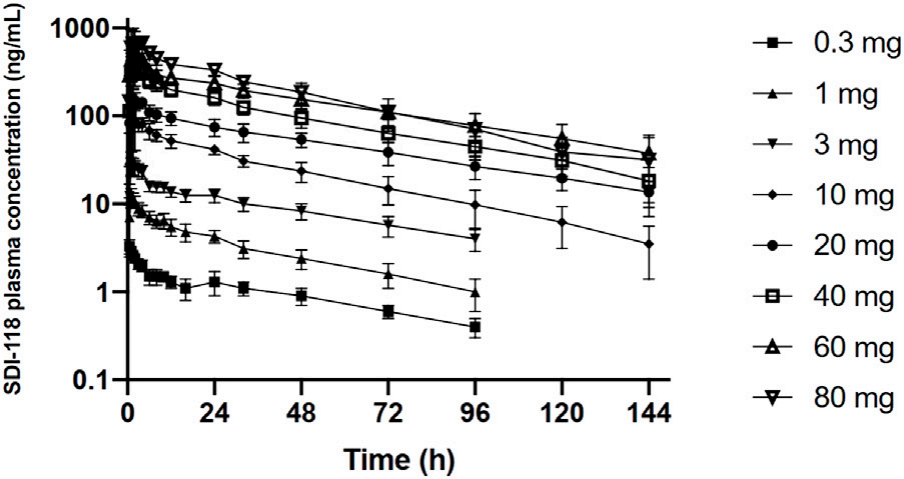
Graphic representation of the (log) plasma concentration over time profile of the single ascending dose part of the study. The graph demonstrates dose proportionality over the entire dose range.

PK data were analyzed in the subjects of Part A (n = 16). SDI-118 was rapidly absorbed in fasting condition (median T_max_ varied from 0.5 to 2.75 h) with a trend for increasing T_max_ values with increasing dose. The disposition of SDI-118 was multiphasic. SDI-118 was eliminated slowly with the terminal elimination phase characterized by a mean apparent elimination half-life that varied between 33.8 and 49.9 h among doses. There was no apparent effect of dose on half-life. The exposure to SDI-118, as based on C_max_ and AUC, increased dose proportionally. Values for the geometric mean C_max_ ranged from 3.42 (0.3 mg) to 854 (80 mg) ng/mL and those for AUC_inf_ from 120 (0.3 mg) to 25,500 (80 mg) ng*h/mL. PK results are displayed in Figure 1. A statistical analysis of this data indicated that the pharmacokinetics of SDI-118 are dose-proportional over the entire dose range investigated (0.3–80 mg) as based on AUC_0-last_ and AUC_inf_. Pharmacokinetics were slightly less than dose-proportional for C_max_, but the deviation from dose proportionality was minimal (approximately 5%). The interindividual variability of SDI-118 pharmacokinetics was low to moderate (in the range of 15%–30%).

**FIGURE 2:**
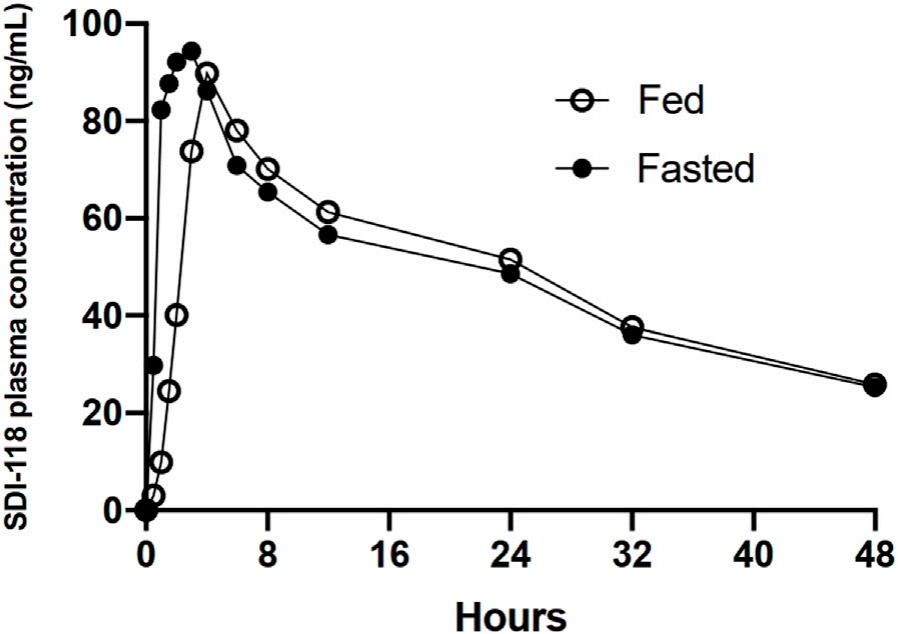
Graphic representation of the plasma concentration over time profile of 10 mg of SDI-118 compared between the fasted and fed state. There was no significant difference in exposure based on C_max_ and AUC. T_max_ was delayed from 1.5 h on average in the fasted state to 4 h on average after a standardized high fat meal.

No clinically relevant effect of food on the extent of exposure (C_max_, AUC_0-last_, AUC_inf_) was detected on the PK analysis set in Part C (n = 8). The median T_max_ was delayed in the presence of food from 1.5 h in fasting state to 4.0 h (min 3.0 to max 6.0 h) in fed state. The same statistical analysis was performed on the PK completers set (n = 7). No significant changes in results were observed. Results of food effect are displayed in Figure 2.

### Pharmacodynamics

Data from all subjects in Part B (n = 8) were included for all target occupancy, PK/PD, and fMRI assessments.

**FIGURE 3:**
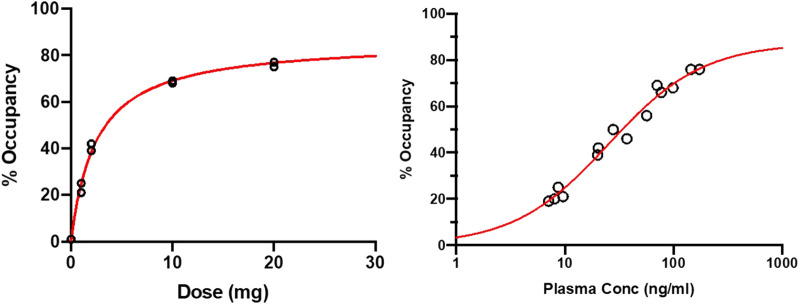
Target occupancy by plasma concentration and dose based on ^11^C-UCB-J PET measurements around T_max_ and 24 h post-dose (left) Dose-occupancy relationship at T_max_ (right) (log) plasma concentration-occupancy relationship at T_max_ and 24 h post-dose excluding the 24 h post-dose data from the lowest dose level (i.e. 1 mg). The EC_50_ lies around 25 ng/ml. Plasma level of ∼200 ng/ml are predicted to render ∼90% of maximal occupancy.

**FIGURE 4:**
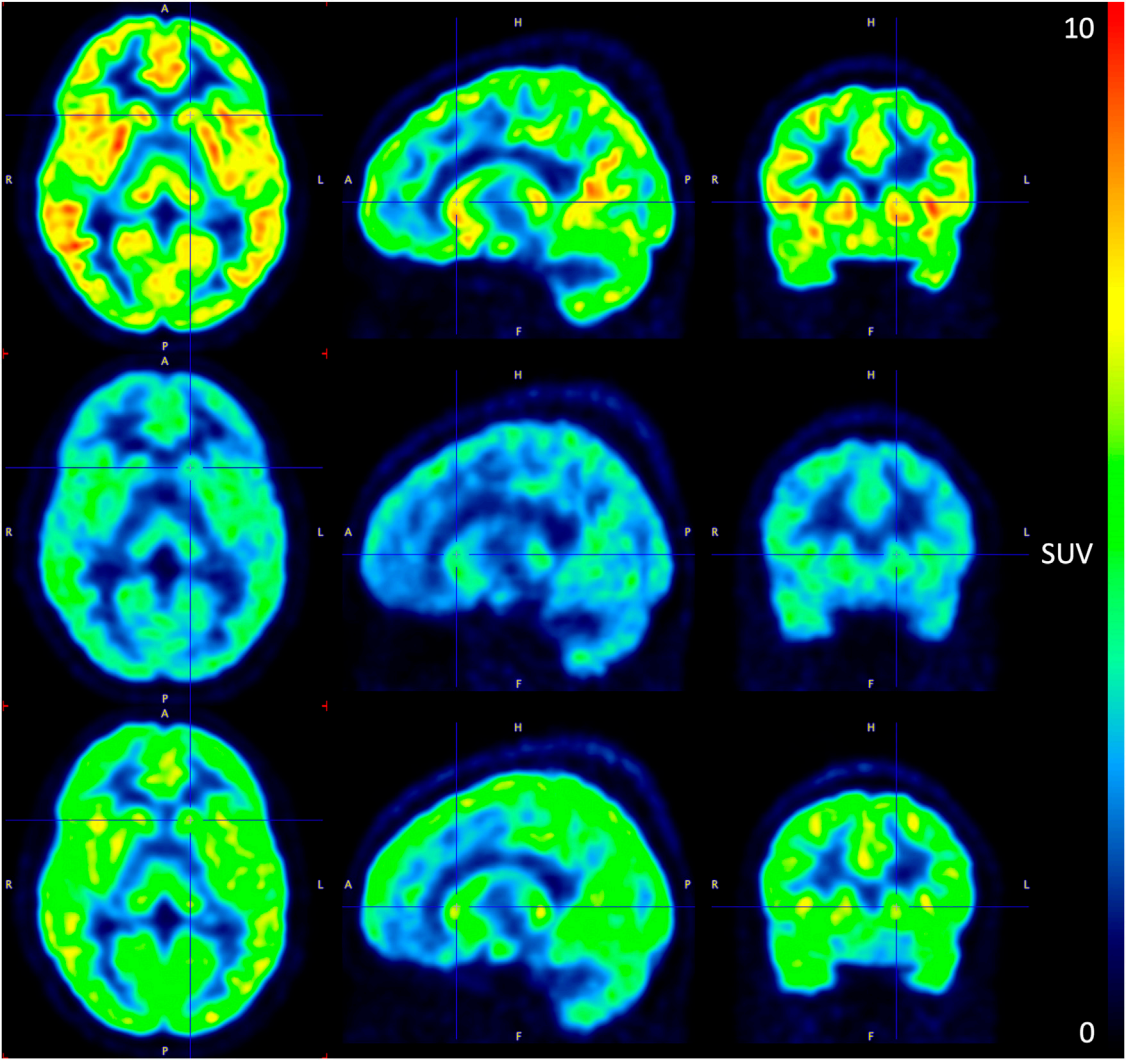
PET image of ^11^C-UBC-J binding during the pre-dose (top), T_max_ (mid), and 24 h post-dose (bottom) scan at the 10 mg SDI-118 dose level in a selected subject (left) axial plane (mid) sagittal plane (right) coronal plane. SUV: standardized uptake value.

At baseline, the regional volumes of distribution of [^11^C]-UCB-J in the brain were similar to what was reported in a recent study using the same radioligand. (Koole et al., 2019). After oral administration of SDI-118, a dose- and concentration-dependent increase in SV2A occupancy was observed with the 1TC global method. Mean SV2A occupancies around T_max_ were 23%, 41%, 69%, and 76% after administration of 1 mg, 2 mg, 10 mg, and 20 mg of SDI-118, respectively (cfr. Figure 3, left; Figure 4). Peak occupancy (i.e. 76%) was reached around T_max_ for the two subjects tested at the highest dose of 20 mg. Mean occupancy decreased to close to 0%, 20%, 48%, and 61% approximately 24 h after administration of 1, 2, 10, and 20 mg of SDI-118, respectively. SRTM analysis using the centrum semi-oval as reference region showed similar occupancy results as with 1TCM.

For PK/PD analysis PK and PET data collected around T_max_ (i.e. 2 h post-dose) and 22 h post-dose were used. PK samples were collected on these time points and 30, 60, and 90 min after. At the highest dose tested (i.e. 20 mg) that induced a peak SV2A occupancy of 76% around T_max_, the mean plasma concentration of SDI-118 was 159 ± 20.5 ng/ml. The differences observed in regional volume of distribution values were low and within the test-retest variability of the [^11^C]-UCB-J PET tracer. (Finnema et al., 2018). Plasma concentrations of SDI-118 were relatively stable across the four time points. A mixed-effect E_max_ model was fitted to the target occupancy *versus* the observed PK at the beginning of the PET measurements, with E_0_ set to zero. EC_50_ was estimated at 27.3 ng/ml (95% CI: 18.4 ng/ml to 36.3 ng/ml), with an E_max_ of 89.2% (95% CI: 80.1%–98.4%). The EC90 (i.e. the concentration where 90% of the maximum effect is reached) of the SV2A occupancy is 245 ng/ml and is predicted to be exceeded around T_max_ with a SDI-118 dose of approximately 40 mg. A dose of 10 mg once a day is predicted to maintain a mean SV2A occupancy of 76% at trough level at steady state. An additional E_max_ model analysis was performed excluding the 22-h post-dose data from the two subjects in the lowest dose level (i.e. 1 mg) treatment group because receptor occupancy at that time point was not reliable (close to or at the limit of detection). Additionally, metabolite analysis at 22 h post-dose for these subjects was considered as not reliable enough to accurately estimate the regional volume of distribution and therefore the global target occupancy at this time point. However, when excluding these two data points, the model parameters are not significantly impacted: EC_50_ was estimated at 28.2 ng/ml (instead of 27.3) and E_max_ at 90.3% (instead of 89.2) (cfr. Figure 3, right).

**FIGURE 5:**
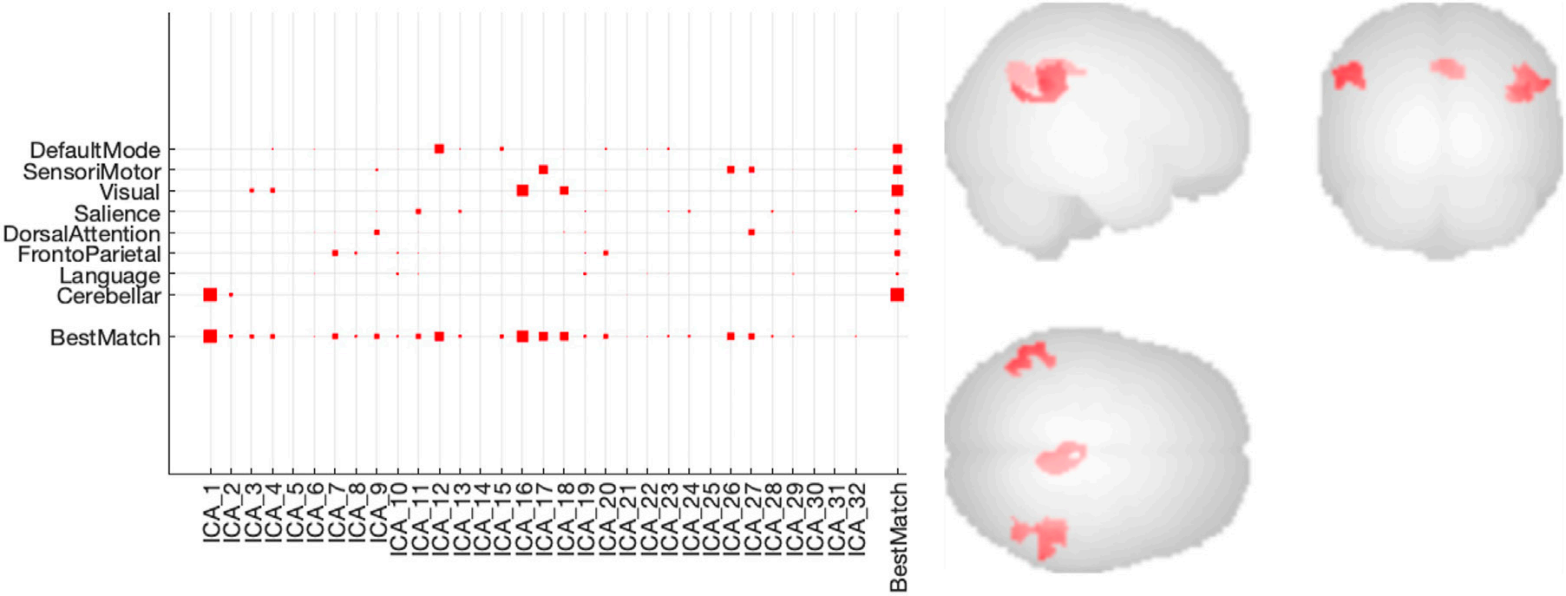
Connectivity results of group-ICA analysis of the rs-fMRI data (left) Degree of spatial match of the different independent components with predefined functional networks (right) Increased functional connectivity within the Default Mode Network (DMN) using a seed based connectivity analysis with IC12 (DMN) nodes as seed regions.

From the 32 independent components resulting from the group-ICA of the fMRI data, IC12 (corresponding to the DefaultMode network or DMN), IC16 and IC18 (Visual network), IC17, IC26 and IC27 (SensoriMotor network), and IC1 (Cerebellar network) could clearly be identified with a network and were considered seed regions for a seed-based connectivity analysis (cfr. Figure 5, left). This analysis revealed a significant increase in connectivity after dosing within the DMN when comparing baseline conditions with all dosing conditions (cfr. Figure 5, right). In terms of a dose-dependent effect, a higher difference in beta parameters for the different subjects could not be observed within the DMN for the higher dose conditions compared to the lower dose conditions at T_max_.

Changes from baseline in BL-VAS and POMS were compared between each active dose and placebo at each time point during the study. No consistent statistically significant changes from baseline, nor apparent trends were observed for either questionnaire.

## Discussion

The incidence of disorders involving cognitive dysfunction is increasing worldwide. This, and the lack of novel pharmacological alternatives for their treatment, necessitates the testing of treatment modalities with novel mechanisms of action. Enhancing the efficiency of synaptic transmission through modulation of synaptic vesicles in the presynaptic terminal has shown to enhance cognition in preclinical studies. In this phase one randomized controlled trial the safety and tolerability of single ascending oral doses of SDI-118, a novel modulator of SV2A, was investigated along with its pharmacokinetic profile and target occupancy.

Administration of SDI-118 as a single oral dose up to 80 mg was found to be safe and well tolerated in healthy male subjects. There were no SAEs or adverse events leading to subject discontinuation from the study. All drug related TEAEs were mild in intensity. The most frequently reported drug related TEAEs were dizziness, hypersomnia, and somnolence, the latter reported only at the highest dose tested (i.e. 80 mg). The onset time of these events was generally short after dose administration, and their duration was usually short with resolution on the same day of dosing or the day after at the most. These mild and transient side effects started at the 10 mg dose and were also observed at higher doses. The 10 mg dose gave a measured occupancy of close to 70%, which translates to 76% of the predicted E_max_ (89.2%). Occupancy at the higher doses would then progressively increase to a maximum of 96% of the predicted maximum at the 80 mg dose. Thus, the observed mild and transient side effects could be consistent with a target related effect seen, in some individuals, at occupancy levels greater than ∼60%. An off-target effect cannot be completely ruled out, although in a screening panel of receptors and enzymes no activity was observed up to a concentration of 10 µM. Further studies will elucidate this. None of the observed clinically significant changes in vital signs, laboratory parameters, and ECG intervals during the study were considered related to the administration of SDI-118. The intended dose range (cfr. infra) is significantly lower than the range in which the observed events were reported in the study, and SDI-118 was very well tolerated in the intended dose range.

After oral administration of SDI-118 as a capsule in fasting condition, SDI-118 is rapidly absorbed with a median T_max_ of 1.5 h. The pharmacokinetics of SDI-118 were dose-proportional over the entire dose range tested. The apparent mean terminal elimination half-life of SDI-118 varied from 34 to 50 h over the dose range tested. The interindividual variability of SDI-118 pharmacokinetics was considered as low to moderate (in the range of 15%–30%). The administration of food (as a standardized high fat meal) did not affect the extent of exposure to SDI-118. After food intake, the median T_max_ of SDI-118 was delayed to 4 h. The long elimination half-life and the lack of a substantial food effect could allow for daily dosing without restrictions on the timing of dosing.

The dose-dependent inhibition of [11C]UCB-J binding to SV2A by SDI-118 is consistent with a very good brain penetration and target engagement of SDI-118 with an EC50 of 27.3 ng/ml (95% CI: 18.4 ng/ml to 36.3 ng/ml) and an Emax of 89.2% (95% CI: 80.1%–98.4%). This Emax is similar to that achieved with levetiracetam and brivaracetam using the same PET ligand. (Finnema et al., 2019). A single dose of approximately 40 mg is predicted to give concentrations above the EC90 at Tmax. A dose of 10 mg once a day is predicted to maintain a mean SV2A occupancy of 76% at trough level at steady state.

A group ICA-driven and seed-based analysis of the resting state BOLD fMRI data showed increased functional connectivity within the nodes of the default mode network after dosing. Core regions (nodes) of the default mode network are the left and right hippocampi, the medial prefrontal cortex, the posterior cingulate cortex (PCC) and the bilateral inferior parietal lobes (IPL). (Buckner et al., 2008). Here we observed increased functional connectivity mainly between the latter three nodes (PCC and left and right IPL) at T_max_ compared to baseline. This effect was not dependent on the dose level in this small sample size. The default mode network is implicated in cognitive processing, notably mind-wandering, introspection, future thinking, and autobiographic memory. (Buckner and Carroll, 2007; Buckner et al., 2008). Moreover, default mode network disruptions have been reported in an array of neuropsychological disorders, including Alzheimer’s disease. (Lustig et al., 2003; Greicius et al., 2004; Hafkemeijer et al., 2012; Grieder et al., 2018). In the latter, progression is associated with reduced default mode network functional connectivity. Further work is necessary to study the role of SDI-118 on the default mode network connectivity and associated cognitive effects in dementia.

In conclusion, the beneficial safety and pharmacokinetic profile of SDI-118, as well as the results of the target occupancy measurements warrant further clinical exploration of this compound. As is the case for most early phase trials, SDI-118 was tested in a limited number of subjects with no significant comorbidities, and for a short period of time. This could potentially limit the external validity of this study. The generalisability of the results should therefore be verified in subsequent clinical trials in selected target populations suffering from cognitive disorders.

## Data Availability

The raw data supporting the conclusion of this article will be made available by the authors, without undue reservation.
